# Supplementary material for: Multiplex model of mental lexicon reveals explosive learning in humans
Source: Sci Rep. 2018 Feb 2;8:2259. doi: 10.1038/s41598-018-20730-5 (PMC5797130; doi:10.1038/s41598-018-20730-5)
Supplement: Supplementary file 1 — Supplementary Information [file 41598_2018_20730_MOESM1_ESM.pdf]

Supplementary Information for “Multiplex model of mental lexicon reveals explosive learning in humans”

M. Stella, M. N. Beckage, M. Brede and M. De Domenico

This is the supplementary material for the main manuscript and it is structured in the following sections:

1. Topology of Individual Layers;
2. Structural Irreducibility Analysis;
3. Degree Correlations and Corrections;
4. Reshuffling null models;
5. Attribute Correlations;
6. Overlap Between Individual Largest Connected Components;
7. No Explosive Transitions in the Growth of Largest Connected Components;
8. No Explosive Transitions in the Growth of the Intersection of Largest Connected Components;
9. Frequency Gap in the Partial Reshuffling Null Model;
10. Motivation for Coupling the Phonological Layer with other Semantic Layers
11. Coupling Phonology and Semantics leads to Explosivity
12. A Note about Polysemy and Polysemy Scores
13. On the Interaction Effect “Frequency + Concreteness”
14. Monte Carlo Sampling for Lexical Experiments
15. A Reduced Growth Model Induced by Concreteness Scores
16. A Distance for Quantifying the LVC Growth Across Different Models

1. Topology of Individual Layers

The Supplementary Table S1 contains different network metrics for the individual layers of the **multiplex lexical representation (MLR)** described in the main text. The reported network features are: mean degree $\langle k \rangle$, mean local clustering coefficient *CC*. size of the largest connected component |LCC|, network diameter D and mean shortest path length $\langle d \rangle$.

In the MLR, degree counts the number of similarities a given word has on a given layer [1]. For instance, on the association layer a word with degree 5 has exactly 5 words associated to it. On the phonological layer, the degree coincides with its phonological neighbourhood size [2]: a word with degree k has exactly k similar sounding words. Words with higher degree in semantic networks were found to have lower age of acquisition norms [3], while degree on the phonological layer correlated positively with word confusability [4]. The main cognitive intuition of degree as a network metric is that words with multiple semantic/taxonomic associates or multiple phonological neighbors are indeed important in the lexicon as they can be accessed through a variety of links by mental navigation of the lexical pathways. As reported in Supplementary Tab. S1, the layers of free associations and taxonomic relationships have higher mean degree compared to the layers of synonyms and phonological similarities.

The mean local clustering coefficient *CC* measures how much, on average, neighbourhoods of nodes resemble complete graphs [1]. The mean clustering coefficient *CC* is the mean local clustering computed across all nodes of a single-layer network. Local clustering on the phonological network was found to positively correlate with word confusability in lexical recognition tasks [4]: the more locally clustered a word was the easier it was for participants to fail in identifying it. Together with the shortest path length, clustering determines the so-called small-world property [5].

The shortest path length among any two nodes in a single-layer network is defined as the minimum number of network hops necessary for navigating the network from one node to the other. In the multiplex lexical representation we ignore any cost for jumping across layers, hence no inter-layer links are considered. This modelling assumption leads to shortest paths over the multiplex structure coinciding with shortest paths over the aggregate network.

Network	$\langle k \rangle$	CC	$\|LCC\|$	D	$\langle d \rangle$
**Free Associations**	8.7	0.093	6431	7	3.8
**Free Associations CM**	8.7	0.007(6)	6420(10)	8(1)	3.6(2)
**Synonyms**	3.1	0.28	5261	18	6.2
**Synonyms CM**	3.1	0.002(1)	6370(40)	13(1)	5.4(3)
**Taxonomic Relations**	9.9	0.048	7910	11	4.0
**Taxonomic Relations CM**	9.9	0.014(7)	7930(30)	8(1)	3.6(2)
**Phonological Similarities**	3.6	0.34	3668	22	6.7
**Phonological Similarities CM**	3.6	0.005(1)	4560(30)	11(1)	4.3(2)
**Aggregate Network**	23	0.084	8519	8	3.3
**Aggregate Network Random**	23	0.014(7)	8520(10)	7(1)	3.1(1)

Supplementary Tab S1: Network metrics for the individual layers in the Multiplex Lexical Representation and for randomised null models (configuration models): mean degree $\langle k \rangle$, mean local clustering coefficient CC. size of the largest connected component |LCC|, network diameter D and mean shortest path length $\langle d \rangle$. As null models we chose configuration models, i.e. networks with randomised links preserving the original degree of words in the layers of the MLR. Error margins indicate standard deviations based on 200 random realisations. For instance 8(1) means 8 $\pm$ 1.

As reported in Supplementary Tab. S1, all of the MLR layers are small-world networks. We stick to the established definition of small-worldness from the literature of cognitive networks [3,6] according to which a single-layer network is a small-world if, compared to equivalently sized Erdős–Rényi random graphs, it exhibits a significantly higher mean clustering coefficient and a comparable mean shortest path length. Small-worldness has been found independently in different types of semantic networks [3] and in phonological networks as well [2]. As already suggested in previous works [6,4], small-worldness might be cognitively beneficial to language learning and use, as it might allow for efficient navigation within the mental lexicon. Furthermore, empirical evidence has shown that small-worldness is related to language learning in children [6]: semantic network lexicons of late talkers, who are likely to exhibit language learning deficits, do show small-worldness to a much smaller degree compared to lexicons from children learning words at normative pace.

Let us also report on a few details behind network construction of the layers analysed above. For the sake of simplicity and for comparison with previous works which used free associations from the Edinburgh Thesaurus [7,8], we considered as equally strong all free associations provided by at least two subjects in the original dataset of the Edinburgh Thesaurus. We then made links from directed to undirected by not considering the directionality and neglecting differences between indegree and outdegree for reasons of simplicity, similarly to what other studies do when dealing with free associations [9,10].

For the phonological layer no threshold is considered, as the measure of phonological similarity has been established in the field by Luce and Pisoni [11] as a common way of establishing which words sound similar to each other. This measure considers differences of one phoneme only and it is highly predictive in terms of several linguistic tasks [4,11,12,13].

Are these four layers representative enough? Already the free association layer contains different types of word relationships and has therefore a multi-relational nature. Previous literature underlined how powerful free associations can be in tasks such as quantifying the creativity of people [9] or identifying writing styles [8] or also predicting early word acquisition in young toddlers [14] . Because of these successful applications, we considered free associations as being one of the most fundamental layers to consider in a multiplex representation. However free associations lack any ordered structure, as they do not represent links of a specific type. This is why we also considered synonyms, where links are defined as indicating overlap in meaning and have been shown to be of great importance for investigating cores in language by previous independent studies [3,15,16].

There is extensive evidence indicating that taxonomic relationships do influence both word acquisition and language use in several different tasks [17], this is why we considered also the taxonomic layer in our analysis.

Since the above three layers consider semantic features of words, we then wanted to associate them with other aspects of language where it was easy to define links among words. We did not choose word co-occurrences as in the literature there are several indications that even up

to 50% of links in co-occurrence networks can be spurious associations rather than syntactic relationships [18]. Our choice then fell on phonology, where we used the measure of phonological similarity by Luce and Pisoni [11], which was later used by Vitevitch for the creation of phonological networks (cf. [2]).

2. Structural Reducibility Analysis

Multiplex networks are a natural framework for considering multi-relational interactions within a single representation [19]. Nonetheless, it is important to quantify model parsimony: does including different layers or relations give additional information compared to an aggregated view? Aggregating here means considering an "aggregate" network where edges are present if they appeared in any of the individual network layers. Structural reducibility analysis [20] investigates whether a given multiplex representation could be aggregated or not, identifying the presence and extent of redundant topological patterns across layers. Structural reducibility analysis relies on: (i) identifying layers similar in their topologies, (ii) aggregating layers if appropriate, and (iii) comparing the richness in topological patterns of the aggregated multiplex layers against the aggregated network, obtained by projecting all the links in the multi-layer structure on a single-layer network. Analogously to a hierarchical clustering, the procedure identifies similarity of layers and quantifies how distinguishable the multiplex is against aggregate versions of two or more layers. The whole procedure is based on the Von Neumann entropy of each multiplex layer and we refer to [20] for further details.
In order to assess the benefit of the multiplex representation, we adopt the procedure suggested in [20]. Results are reported in Supplementary Fig. S1. Interestingly, all the three semantic layers are clustered together and are only aggregated with the topological layer at the last stage of the hierarchical clustering. At every level of the hierarchy a relative entropy measure is reported, expressing how different is the average entropy of the considered layers compared to the aggregated multiplex network with all layers collapsed into one network. The higher the relative entropy, the more different the multiplex layers are, on average, compared to the aggregated single-layer network. Aggregating layers with different topological structure might cause a loss of information. In fact, after the aggregation, one would consider as indistinguishable (i.e. coming from the same layer) two classes of links that differed greatly in their assortment among nodes and were thus, in the multiplex representation, distinguishable (i.e. coming from different layers). Notice that the ideas of distinguishability and mixing among links coming from different layers relates to the quantum mechanics machinery behind irreducibility analysis [20]. Beyond the technical details, it is the information loss when aggregating multiplex layers that can indeed motivate pursuing a multiplex representation rather than an aggregated one. As reported in Supplementary Fig. S1, the highest information loss is relative to aggregating the original MLR with all its 4 layers kept as distinct. This result indicates that no aggregation can be performed without losing topological information on the pathways among words, thus

motivating the importance of a multiplex representation of the mental lexicon over the 4 aspects we considered.

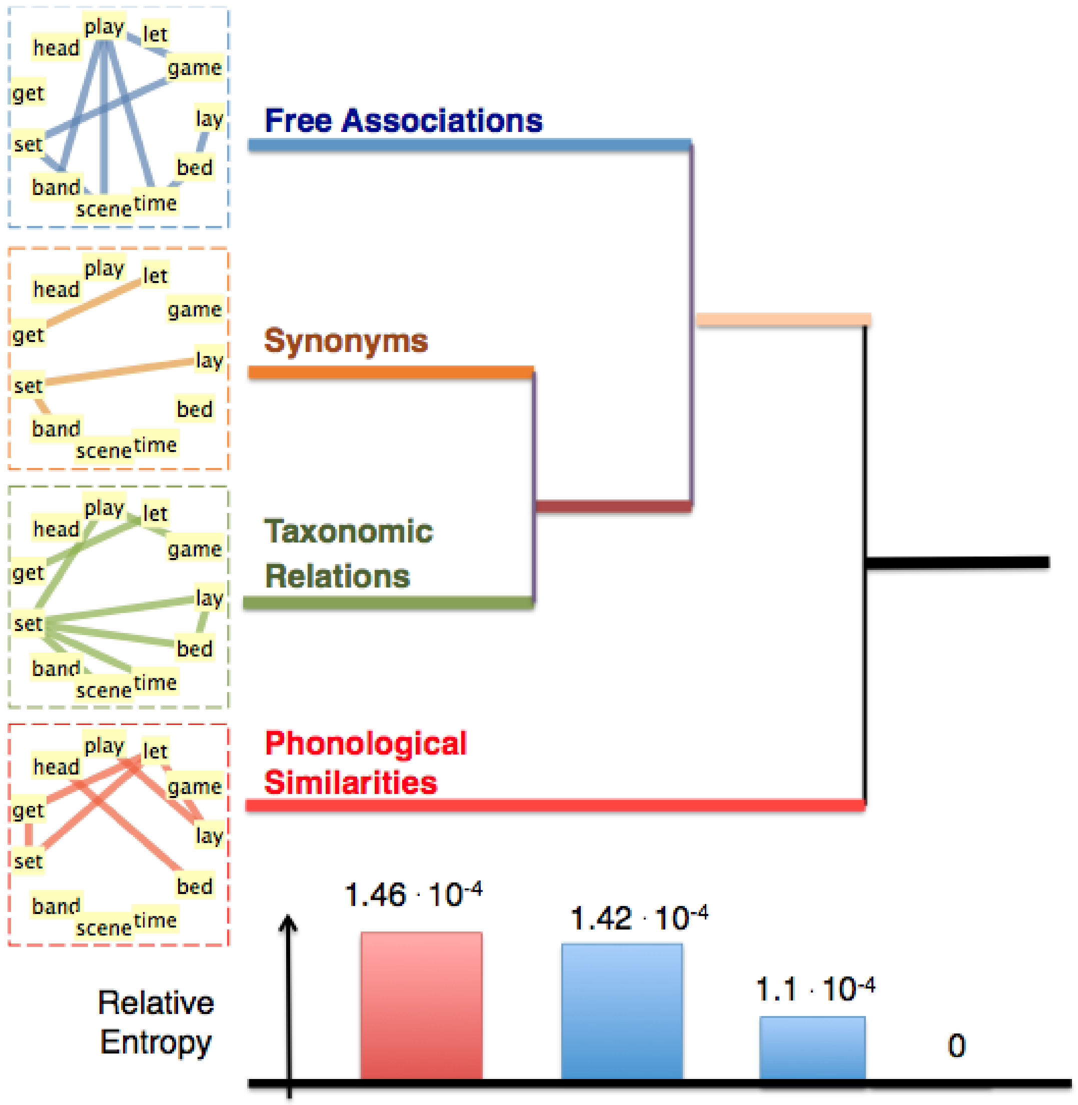

Supplementary Figure S1 : Structural reducibility analysis of the different layers in the multiplex lexical representation of the mental lexicon. Layers are hierarchically clustered according to the similarity in their topologies. At every level of the resulting dendrogram, an entropy measure quantifies how much more topological information a certain collection of layers has compared to the case in which all layers are projected into a single-layer network only (i.e. the aggregate network). The configuration having most topological information compared to the aggregate is the one in which all the layers are kept as separate: our MLR cannot be aggregated or clustered without losing topological information on the patterns among words.

3. Degree Correlations and Corrections

As already discussed in SI Sect. 1, the degree of words in linguistic networks encapsulates some information about the cognitive and linguistic patterns recurring in word processing or acquisition. For instance, Stella et al. [14], found that degree of words in the free association

layer of young children was a powerful predictor of word learning trajectories in the early phases of development. On the phonological level, degree corresponds with the phonological neighborhood size, which correlates with word confusability and retrieval time in lexical decision tasks [4].
It is therefore expected for degree to correlate with other linguistic node attributes such as the ones tested in the main paper. We report such correlations in Supplementary Tab. S2. As a degree measure on the whole multiplex network we consider multidegree, i.e. the sum of the individual degrees across layers (cf. [21]). Positive (negative) correlation values of Kendall Tau indicate that higher degree implies higher (lower) node attribute.

Degree Correlations	***Free Associations***	***Synonyms***	***Taxonomic Relations***	***Phonological Similarities***	***Multiplex Ntw. (Multidegree)***
**Frequency**	0.43	0.22	0.28	0.22	0.42
**Age of Acquisition**	-0.37	-0.16	-0.19	-0.25	-0.33
**Polysemy**	0.29	0.43	0.54	0.24	0.5
**Concreteness**	0.14	-0.06	-0.06	0.18	0.07
**Reaction Time**	-0.43	-0.20	-0.28	-0.15	-0.39
**Word Length**	-0.24	-0.21	-0.13	-0.62	-0.35

Supplementary Table S2 : Kendall Tau correlations between degree on a given multiplex network layer and node-level attributes (frequency, age of acquisition, polysemy, concreteness, reaction time and word length). The Kendall Tau correlation expresses the fraction of agreements minus disagreements in ordering when words are ranked according to the two tested features (e.g. if word *i* has degree higher than word j, how more likely is it to for word *i* to have a higher attribute than word *j* compared to word *i* having a smaller attribute than word *j*?). Kendall Tau significance tests indicate that all the reported correlations are statistically significant at a 0.05 significance level.

As reported in Supplementary Figure S2, the LVC-out words are in general less connected compared to LVC-in words. One could then test if the differences in node level attributes between LVC-in and LVC-out words are merely an artefact of degree, provided that degree itself correlates with the node level attributes (cf. Tab. S2). Degree corrections allow to sample words with the same degree both in the LVC and outside of it, thus erasing first order differences in local connectedness (see Tab. 1 in the main manuscript). In the main text (cf. Tab. 1), degree corrections for the average node attributes of LVC-out words are performed by considering the degrees on the multiplex layer with the highest correlation to that specific node attribute (see the Monte Carlo Sampling SI Section down below). For instance, frequency correlates the most with word degrees in the association layer, therefore when computing the average frequency of LVC-out words with the same degree distribution as LVC-in words the degrees on the association layer are considered (see Methods of the main text for further details).

Let us underline that in the main text we also compare the distributions of times at which the LVC appears. We numerically checked that in all cases, over the different iterations reported in the main text, the distribution of times at which the LVC appears for a given acquisition model does not display a heavy-tail (as it happens in the frequency distribution, for instance) but rather a (sligthly) stretched exponential distribution, resembling a Gaussian. Resemblance of a Gaussian distribution is tested with a D'Agostino K-squared test, which confirmed that all distributions for different acquisition models were compatible with a Gaussian distribution within a 0.1 significance level. The shape consistency indicated that the distributions all represent distributions similar to the normative one, thus making the choice of the overlapping coefficient a valid one.

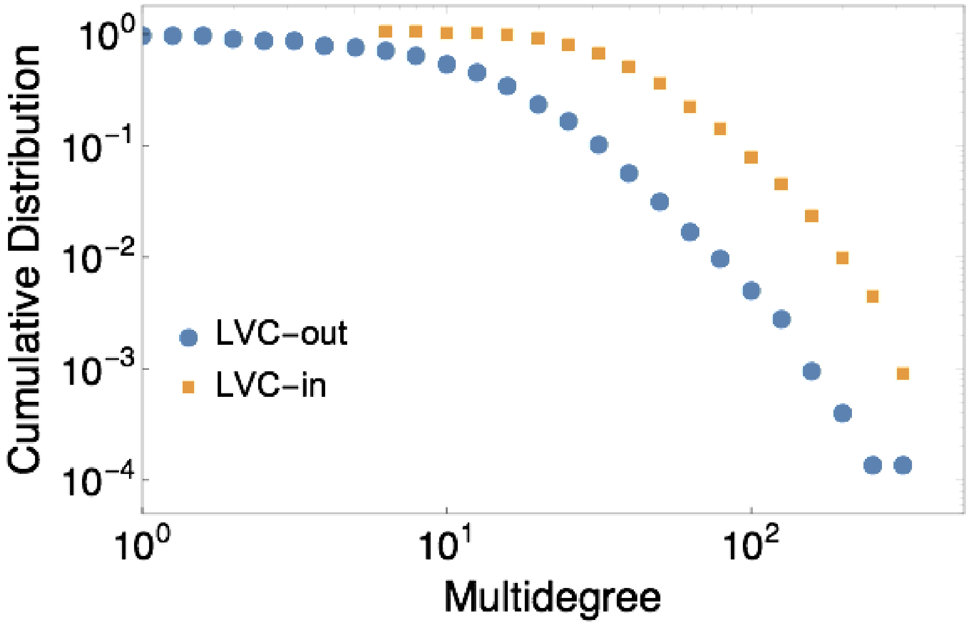

Supplementary Figure S2 : Cumulative probability distribution of finding a node with a multidegree higher than a given value in the multiplex lexical representation for LVC-out words (blue) and LVC-in words. For instance, the probability of finding nodes with more than 100 connections across all layers is $2.5 \cdot 10^{-2}$ outside of the LVC and roughly $1 \cdot 10^{-1}$ within the LVC. Hence, the LVC is richer in higher degree nodes compared to the rest of the multiplex lexical representation.

4. Reshuffling Null Models

Our reshuffling null models disrupt correlations between words within a given layer and across layers mainly in two ways (see also Methods in the main text):

1. *partial reshuffling* keeps words aligned with their topological position but it reshuffles individual node-attributes, i.e. it makes individual *rows* of Table S2 close to 0 when performed on all the layers;
2. *full reshuffling* performs a reshuffling of word labels, thus destroying any correlation between topology and node attributes, i.e. it makes the *whole* Table S2 close to 0 when performed on all the layers.

A visualisation of the different reshuffling models used in the main text is reported in SI Fig. S3. Notice how both the models preserve the link structure across layers but change the labels or the node attributes at the endpoints. As evident from SI Fig. S3, full reshuffling does not change the node-level attributes a given word has, since the word label and all its features are reshuffled together (see the arrow in SI Fig. 3 (b)). Instead, partial reshuffling allows to consider reshuffled configurations where a given word is attributed another attribute (see the red arrow in SI Fig. 3 (c)).

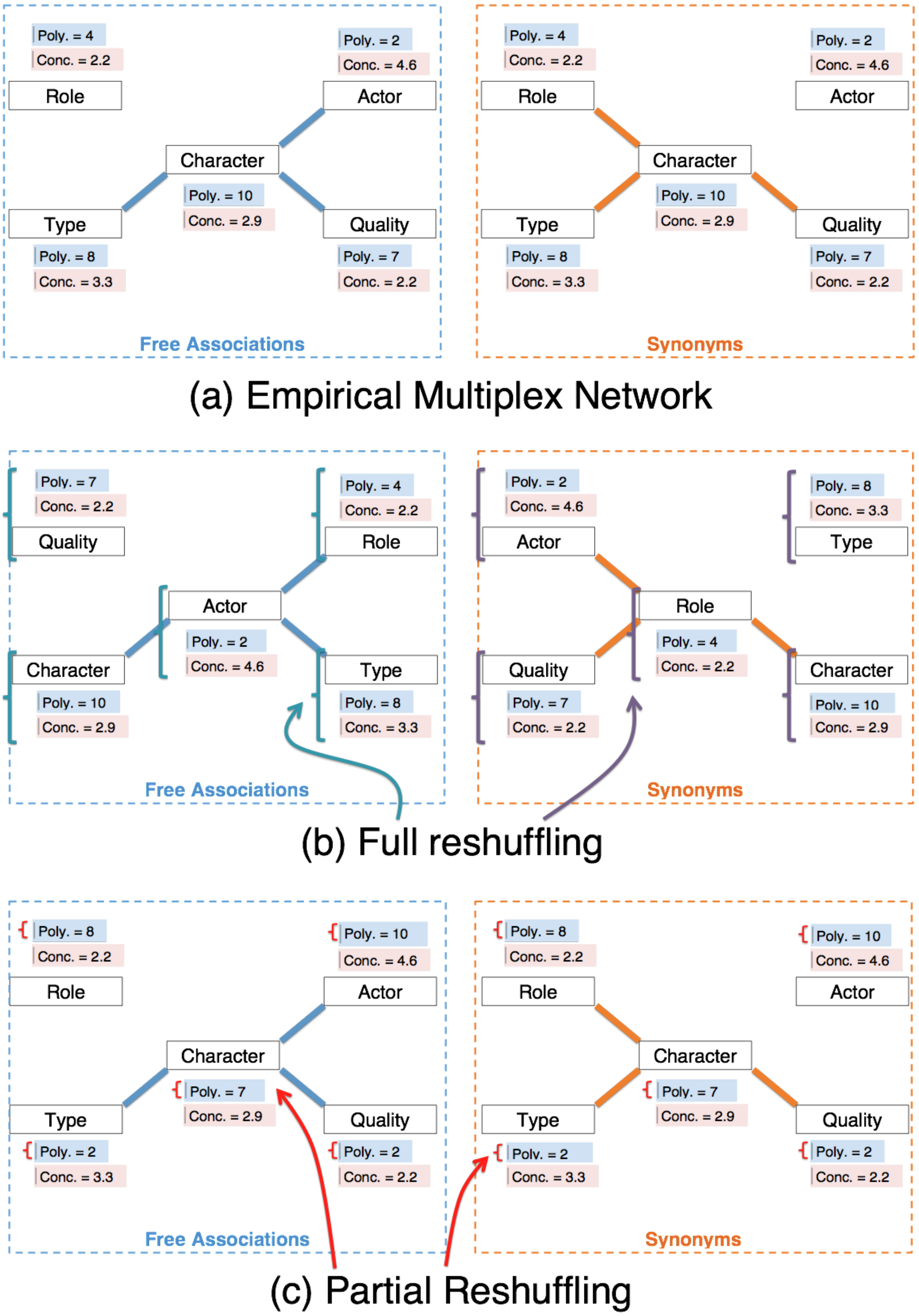

Supplementary Figure S3 : Examples of the reshuffling models for a subgraph of the multiplex lexical network limited to 5 words, 2 layers and 2 node-attributes (polysemy and concreteness). (a): Empirical network. (b) Full reshuffling of word labels, independently on each layer. Polysemic links are mostly disrupted. Arrows of different colour indicates that words are reshuffled independently on every layer (c) Partial reshuffling of node-attributes (e.g. polysemy counts), aligned across layers. Arrows of the same colour indicate that node attributes are reshuffled but kept consistent for a word across all layers. In both (b) and (c), the layout of links is untouched and degree distribution is preserved.

In the main text we performed full reshuffling of either high-polysemy words or of the same number of low-polysemy words. High-polysemy words were defined according to the distribution of polysemy counts in the MLR, which is reported in Supplementary Figure S4. High-polysemy words are those in the heavy tail of the distribution, starting at polysemy count 5 (i.e. words with strictly more than 5 different context-dependent meanings in the dataset). Low-polysemy words were defined as the remaining words (i.e. words with less than 5 different context-dependent meanings in the dataset).

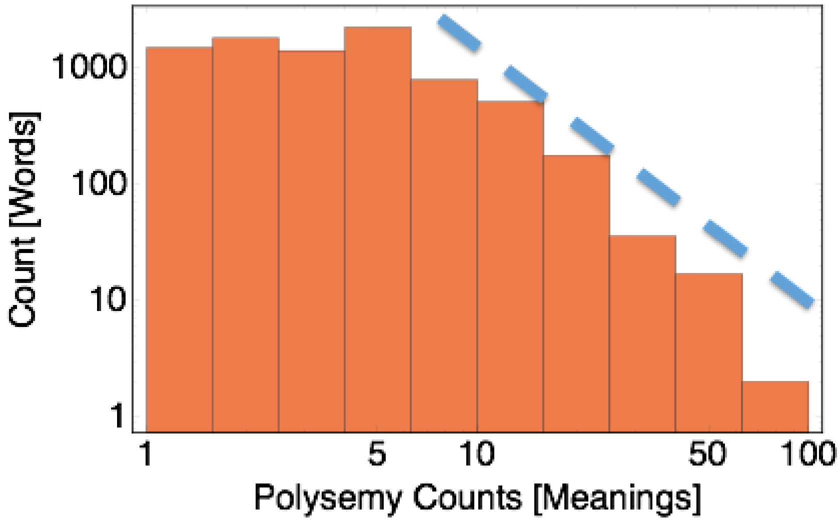

Supplementary Figure S4: Log-log plot of the distribution of counts of words with a given polysemy count (i.e. how many words in the MLR have polysemy count 1, 2, 3, etc.). The dashed blue line is merely highlighting the heavy tail of the distribution, which includes words having more than 5 different meanings in the Wolfram's dataset WordData. No power-law fitting was performed on the data.

5. Attribute Correlations

Partial reshuffling evidently destroys also correlations among node-level attributes of a given word. Supplementary Tab. S3 contains the Kendall Tau correlations among node-level attributes used in the main text.
Notice that in our investigation, we also tested word valence from [22] but we did not find any statistically significant difference, at 0.95 confidence interval, between the valence of words within the LVC and outside of it (p-value 0.35). We got the same negative result with dominance and arousal. This suggests that the LVC structure does not differentiate between affection levels to individual words: words with higher and lower valence are equally mixed within and outside of the LVC structure.

6. Overlap Between Individual Largest Connected Components

In the main text we focus on the largest viable cluster, whose definition for single-layer networks coincide with the one of largest connected component. The largest connected

component (LCC) of a given network is defined as the largest set of nodes that can be reached from each other (Newman, 2010). Analogously to what we do in the main text for distributions of LVC emergence times, we define the *percentage intersection* as the intersection of word lists being in two largest connected components normalised by the maximum intersection possible (i.e. the largest connected component of the aggregated multiplex network). A percentage overlap of 50% between two layers means that half the words in the LCC of the aggregated multiplex network fall in the LCC of both the layers.

Attribute Correlations	***Frequency***	***Age of Acquisition***	***Polysemy Scores***	***Concreteness***	***Reaction Time***	***Word Length***
Frequency		-0.47	0.36	0.08	-0.54	-0.22
Age of Acquisition	-0.47		-0.26	-0.28	0.44	0.24
Polysemy Scores	0.36	-0.26		0	-0.35	-0.21
Concreteness	0.08	-0.28	0		0	0
Reaction Time	-0.54	0.44	-0.35	0		0.12
Word Length	-0.22	0.24	-0.21	0	0.12	

Supplementary Table S3 : Kendall Tau correlations between node attributes used in the main text. Correlations with p-value above a significance level of 0.05 were considered equivalent to 0 (i.e. statistically independent) within a Kendall Tau significance test and thus reported as "0" in the above table.

Notice that the LVC will always be either equal or smaller in size to the smallest pairwise percentage intersection. Pairwise intersections among largest connected components on individual layers are reported as percentage intersections in Supplementary Fig. S5. Full boxes report intersections among empirical layers while empty boxed indicate the reference value for configuration models.

The phonological layer is the layer overlapping the least among the four in the multiplex lexical representation. In all the possible cases, the phonological layer displays smaller intersections with other layers compared to random expectation. These findings agree with the results from the structural reducibility analysis in providing the idea that the phonological layer greatly differs in terms of pathways against all the other semantic layers in the MLR. Differently put, the above topological patterns confirm that phonological similarities among words do indeed look different from either semantic or taxonomic relationships. Our multiplex lexical representation naturally allows to keep phonological word-word interactions as separate from the others.

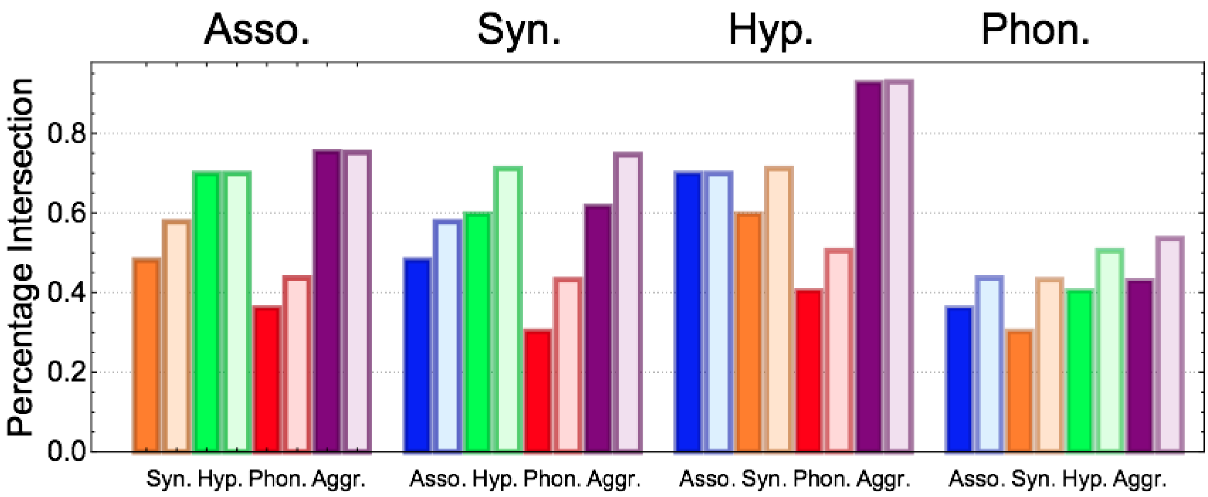

Supplementary Figure S5 : Percentage intersection between largest connected components of different layers ("Asso." for free associations, "Syn." for synonyms, "Hyp." for taxonomic relations and "Phon." for phonological similarities). The aggregated multiplex network is identified with "Aggr.". The plot can be read from top to bottom, e.g. at the top the Asso. layer has a LCC overlapping with the Phon. LCC (bottom) for 35% of the maximum possible value. Empty boxes are relative to configuration models, i.e. randomly rewired networks preserving word degrees. Error bars are of the same size of the box edge.

7. No Explosive Transitions in the Growth of Largest Connected Components

Contrary to what happens in the emergence of the LVC, the growth over time of largest connected components does not display any explosive behaviour. Considering 200 iterations where words are inserted in the multiplex lexical network according to their smeared age of acquisition, none of the simulated word trajectories exhibits an explosive discontinuity in the size of the largest connected component for any of the four individual layers.

Also the connected component of the whole multiplex network (i.e. the connected component of its aggregate [21]) does not display any explosive transition. Average behaviours are reported in Supplementary Fig. S6.

The above numerical results indicate that the explosive emergence of connected structures across all layers, such as the LVC, are indeed distinct topological patterns, different from those exhibited by other connected structures such as largest connected components.

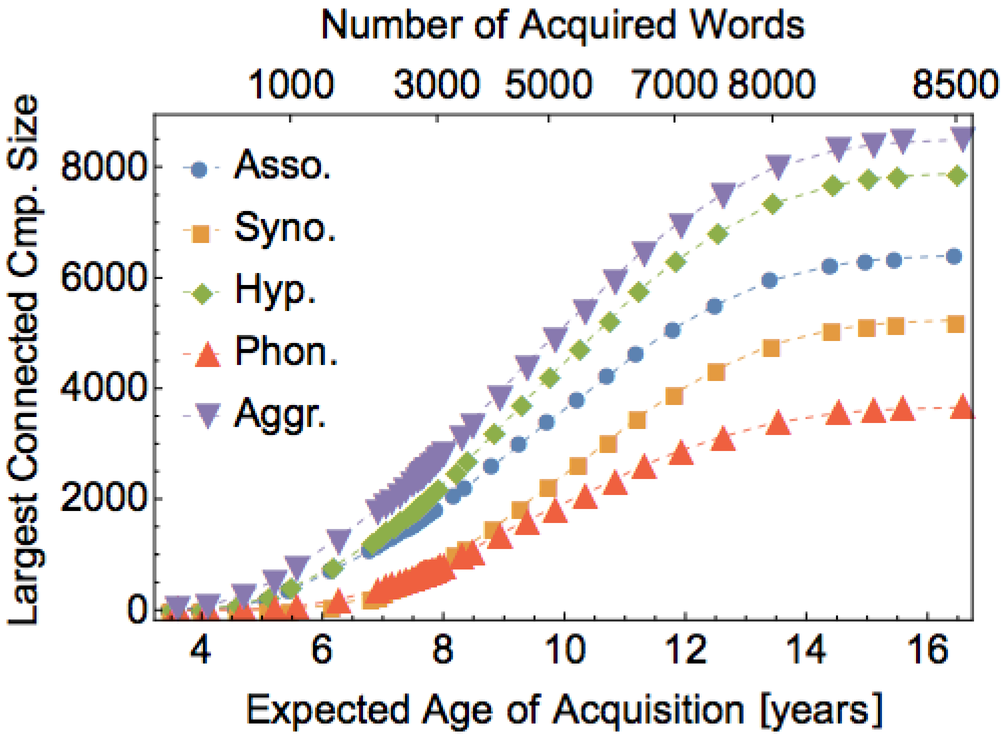

Supplementary Figure S6 : Size of the largest connected component for the individual layers over normative word acquisition ("Asso." for free associations, "Syn." for synonyms, "Hyp." for taxonomic relations and "Phon." for phonological similarities). The aggregated multiplex network is identified with "Aggr.". Error margins indicate standard deviations and are of the same size of the plot markers. Results are averaged over 200 iterations of smeared normative acquisition (in which words are acquired in the same order of their age of acquisition).

8. No Explosive Transitions in the Growth of the Intersection of Largest Connected Components

We want to explore if the explosive pattern present in the LVC is replicated also in other reasonable definitions of cores. As another multiplex measure we explored also the intersection of largest connected components across all layers, which includes 2146 words. Notice that the intersection of LCCs does not require connectedness simultaneously across all layers, i.e. words in the intersection might not be connected with each other by paths using one of type of relationships only. The intersection of LCCs can therefore be considered as having some missing pathways in it, whose potential addition would make it a viable cluster, cf. Supplementary Fig. S7.

Differently from the LVC, the intersection of largest connected components does not display any explosive transition in its size when words are acquired according to normative acquisition (see Methods), neither when individual word trajectories are analysed. Supplementary Figure S8 reports the results for the average case, computed over 200 iterations of smeared normative acquisition.

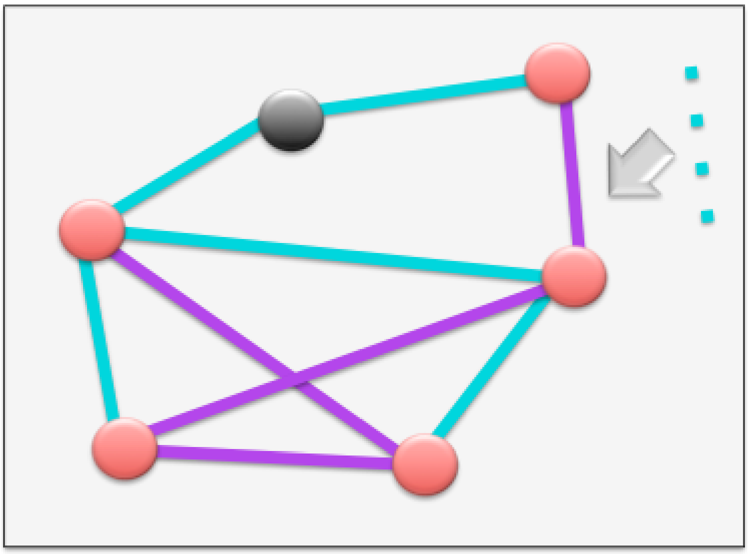

Supplementary Figure S7 : Example network to illustrate differences in the notion of viable clusters and intersection between connected components, with 2 layers (represented by different colours). The intersection of LCCs is highlighted in red. Nodes in the intersection do not constitute a viable cluster as there is one cyan link missing connecting the red node to others by using only links of cyan colours. The addition of this pathway (the dashed link above in the picture) would make the whole intersection a viable cluster. Notice that the 4 nodes in the lower part of the figure do constitute a viable cluster. In general, the LVC is a subset of the intersection of largest connected components.

Even though the intersection of LCCs is indeed a multiplex measure, as it takes into account connectedness on different multiplex layers, it does not require simultaneous connectedness across all layers, as the LVC does instead. The LVC has therefore an additional requirement of viability on *every* layer, with "no holes" in connectedness of the individual layers. Such holes could instead present in the intersection of LCCs. This is the main difference between the LVC and the intersection of LCCs: the former requires viability on every layer while the second does not. Therefore, comparison between the multiplex intersection of LCCs and the LVC points out that it is indeed viability the ultimate cause of explosive behaviour in the multiplex lexical representation.

Notice that, from a cognitive perspective, the stricter requirement of connectedness across all layers allows to navigate across all words of a viable cluster, without having to "jump" across different linguistic aspects. Hence, in terms of mental navigation, the LVC allows for a smaller number of words to be reached from each other within a given aspect of the mental lexicon while the intersection of LCCs might require jumps across different aspects of the lexicon (e.g. between semantic and phonological layers) in order to connect a larger number of concepts.

The absence of explosive behaviour in LCCs and in their intersection further supports the idea of the distinctness of the LVC emergence as presented in the main text.

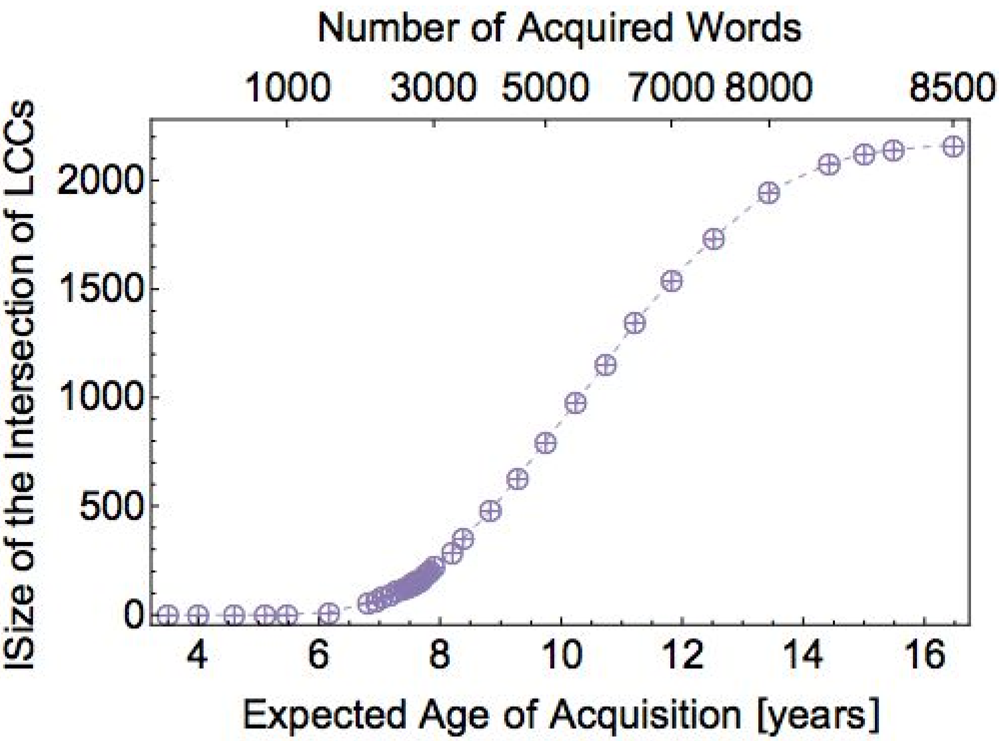

Supplementary Figure S8 : Size of the intersection of largest connected components over normative word acquisition. The aggregated multiplex network is identified with "Aggr.". Error margins indicate standard deviations and correspond to symbol sizes. Results are averaged over 200 iterations of smeared normative acquisition.

9. Frequency Gap in the Partial Reshuffling Null Model

In the main text we quantified the gap in median concreteness and polysemy of words in the LVC when partial reshuffling was applied to words in the MLR. In this section we report the results relative to partial reshuffling of word frequency, presented in Supplementary Fig. S9.

The observed gap between the frequencies in the empirical and in the partially reshuffled models is of similar size to the one observed for concreteness in the main text and it is therefore considerably smaller than the gap observed for polysemy. This suggests that polysemy has a higher influence on the evolution of the LVC compared to either concreteness or frequency of words.

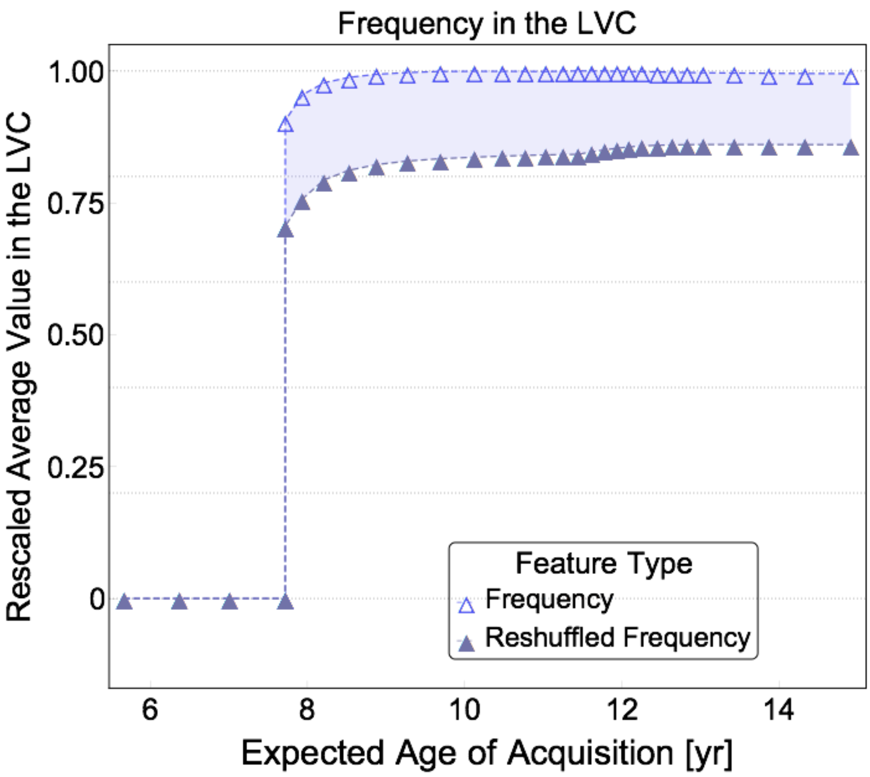

Supplementary Figure S9 : Comparison of average frequency for words in the LVC with normative acquisition in the empirical data and for a partial reshuffling null model. Reshuffling node attributes results in a LVC with reduced frequency. The curves are rescaled by their maximum value and they represent averages over 200 iterations. Error margins are approximately the same size as the dots. We note significant gaps between the empirical and randomised data.

10. Motivation for Coupling the Phonological Layer with other Semantic Layers

While words with larger degrees on semantic networks have been found to lead to more efficient processing or earlier acquisition in several studies (for a review see [23]) the same is not true for the phonological layer, so that a question might rise about the choice of coupling phonology and semantics in investigating models of word acquisition and processing.

Previous independent studies like [24] showed how English words with larger phonological neighbourhoods were more likely to be acquired in toddlers up to month 30. This effect disappears around month 30 of age. At later stages the work from Storkel [25,26] has shown that high neighbourhood density facilitates word learning also in young children before age 7. Starting from age 7, several independent studies indicate that lexical competition effects start inhibiting the facilitation effect in learning new words with more dense phonological neighbourhoods [27,28,29]. Further experiments with young adults [4,13], indicated that more dense phonological neighbourhoods have a positive influence on word memorisation and recollection. While it is not entirely clear how phonological competition emerges, we interpreted the above findings as a positive influence phonological neighbourhoods have on both word acquisition (early in life) and word memorisation (during the adulthood). These

effects motivated our choice of including also the layer of phonological similarities [37] in the multiplex lexical representation of the mental lexicon.

11. Coupling Phonology and Semantics leads to Explosivity

Even though the considered multiplex lexical representation of the mental lexicon is irreducible, it is not clear how individual layers contribute to the explosive appearance of the LVC. In order to test this, we simulated the normative multiplex lexicon growth as in Figure 2 (a) in the main text, but now, to test for the relative importance of individual layers, also consider the case in which one layer is excluded. We then measured the time t* at which the LVC appears and the magnitude D of the explosive transition (i.e. the average number of words within the LVC when it appears). Results are condensed in the following Table:

Removed Layer	t* (years)	D
Free Associations	7.6 ± 0.7	440 ± 60
Synonyms	6.0 ± 0.6	400 ± 50
Taxonomic Relations	7.5 ± 0.7	440 ± 60
Phonological Similarities	No discontinuity is observed	

Supplementary Table S4 : The time t* at which the LVC appears and the average number D of words within the LVC when it appears (i.e. the magnitude of the explosive transition).

We find that, removing either the layer of free associations or taxonomic relationships has minor relevance for the appearance of the LVC. Similar results to what we reported would be obtained if one of these two layers was removed. In contrast, removing the layer of synonym relationships would make the LVC appear at earlier ages and with a slightly smaller discontinuity. More importantly, removing the phonological layer also removes the explosive appearance of the LVC, whose emergence then becomes continuous, without a discontinuous transition. We interpret this finding as a quantitative confirmation that the appearance of the LVC is a novel phenomena arising only when we couple semantics and phonology within the same network framework.

12. A Note about Polysemy and Polysemy Scores

There is not a clear way of defining polysemy in the relevant literature [30]. In particular, Rodd et al. [30] criticised the lack of distinction between polysemy being defined as in “a word having multiple related senses” (e.g. “uniform” distribution and school “uniform”) and polysemy defined as in “a word having with multiple unrelated meanings” (e.g. financial “bank” and river “bank”). While Rodd et al.’s findings indicated that only polysemy in terms

of senses has an influence on lexical processing, an overwhelming number of more recent studies in the last few years [31,32,33] has shown that also a more general definition of polysemy in terms of meanings does impact lexical processing. Because of the above lack of distinction, we prefer quantifying polysemy in terms of polysemy scores, as commonly done in computational linguistics [31,32,33,34].

In the main text, following previous works quantifying polysemy of words in large datasets through WordNet such as [32,33,34] we consider as polysemy scores the number of definitions a word has in the WordData dataset, which coincides with WordNet. This quantification of polysemy scores as entries in a dictionary such as WordNet has been used consistently in [32,33,34].
As an example, from our WordData dataset, for "character" we get the following definitions:

1. {"character", "Noun", "GeneticScience"} -> "(genetics) an attribute (structural or functional) that is \ determined by a gene or group of genes",
2. {"character", "Noun", "Trait"} -> "the inherent complex of attributes that determines a persons moral \ and ethical actions and reactions",
3. {"character", "Noun", "Property"} -> "a characteristic property that defines the apparent individual \ nature of something",
4. {"character", "Noun", "Enactment"} -> "an actor's portrayal of someone in a play",
5. {"character", "Noun", "Recommendation"} -> "a formal recommendation by a former employer to a potential future \ employer describing the person's qualifications and dependability",
6. {"character", "Noun", "WrittenSymbol"} -> "a written symbol that is used to represent speech",
7. {"character", "Noun", "ImaginaryCreature"} ->"an imaginary person represented in a work of fiction (play or film \ or story)",
8. {"character", "Noun", "Adult"} -> "a person of a specified kind (usually with many eccentricities)",
9. {"character", "Noun", "Reputation"} -> "good repute", {"character", "Verb"} -> "engrave or inscribe characters on"}

As a result, "character" has a polysemy score of 9, since it is related to 9 different entries in the WordNet dataset. This polysemy score for "character" includes also the lexical entries related to "font" (entry n. 6) and "nature" (entry n. 8), as reported on page 5. Hence, the polysemy scores analysed in the paper include also homonymy and meanings in different semantic areas.
Notice that we consider all 9 of them mainly because these definitions are deeply related with the structure of the mental lexicon in terms of the links for node "character". As an example, on the layer of free associations "character" is linked to "actor", "book", "disposition", "face", "good", "individuality", "man", "mature", "person", "play", "reference", "sketch", "study", "temperament", "test", "type". All these links can be related to the above definitions of

"character". Additionally, to explain the relationship of character and its associates, we need many if not all of these definitions.

In general, within our analysis, we assume that ALL these links, and hence their related senses, meanings and definitions, are considered in the network structure of the mental lexicon.

13. On the Interaction Effect "Frequency + Concreteness"

It is likely that a combination of node covariates could explain the expected Age of Acquisition, such as a concreteness x frequency interaction. The mechanism allowing for this would be that many high frequency words are abstract and hence have lower concreteness scores while many high-concreteness words are uncommon in language.

We explored this by performing a linear regression analysis of the age of acquisition scores for words for which also concreteness scores and frequency counts were available.

Considering frequency alone provided an R-squared = 0.38 while considering concreteness alone provided an R-squared = 0.005. For comparison, considering polysemy scores only for predicting age of acquisition leads to a model with R-squared = 0.11. These numbers indicate that both polysemy scores and frequency are able to explain more than ten times the variance of age of acquisition scores compared to concreteness.

Considering an interaction term "concreteness x frequency", within a linear model AoA = a * Frequency + b * concreteness, resulted in a considerably higher R-squared = 0.85, with a = 2.05±0.03 (p-value < 10^{-5}) and b = 0.17±0.01 (p-value < 10^{-5}). For comparison, considering a model with "polysemy scores x frequency" leads to a lower R-squared = 0.76.

Hence, the interaction effect "frequency x concreteness" explains the variance in the age of acquisition curve better than the "frequency x polysemy" interaction.

Hence, as expected, coupling frequency and concreteness provides a better model for explaining the age of acquisition distribution as many words that are frequent are also abstract words (i.e. with a low concreteness score). Our results confirm previous studies which showed that concreteness plays a small role in lexical processing together with other linguistic variables (cf. [35]). The same is true also for polysemy: high frequency words tend to be less ambiguous (cf. [36]), hence the increase in R-squared.

While this investigation of the curve of age of acquisition is interesting, notice that in the main manuscript we do not compare frequency, concreteness and polysemy scores by themselves against the age of acquisition scores. We do not try to predict age of acquisition by considering linear models of word features but we rather focus on the dynamics of the LVC. In our models of lexical acquisition, frequency, age of acquisition and the other linguistic variables produce rankings of words. These rankings then determine a growth

dynamics of the multiplex lexical representation. During the growth dynamics we focus our attention on the emergence of the LVC.

Furthermore, in this first exploration of the LVC emergence, we focus our analysis on how individual linguistic features influence the emergence of the LVC, leaving further investigations of interaction terms among different linguistic features as an interesting research question for future work.

However, the above results indicate that when word features are considered individually, then polysemy scores account for age of acquisition considerably more than word concreteness, thus supporting our results from the main manuscript but from a different perspective.

14. Monte Carlo Sampling for Lexical Experiments

Monte Carlo sampling refers to a set of techniques widely used in statistical mechanics for computing efficiently average quantities and moments of distributions from large sample spaces. In network science, Monte Carlo techniques allow to approximate network features of large networks without considering the whole network structure but rather smaller subgraphs [1].

In our case, we adopt a microscopic Monte Carlo sampling for correcting degree biases when selecting sets words within and outside of the LVC. Let us consider comparing the average word length of words within the LVC and outside of it. We could straightforwardly sample all 1132 words in the LVC and all 8531 – 1132 words outside of the LVC, consider their word lengths and then perform the averaging. This procedure would not keep into account the facts that: (i) word length correlates positively with degree, (ii) the LVC contains words with higher degrees. Correlations of word features with degree might influence the differences between LVC-in and LVC-out words observed in the main text. In order to get rid of these correlations and compare samples of the same size (LVC-out words are more than five times the ones in the LVC) we resort to the following Monte Carlo sampling procedure:

1) – Select one word W1 within the LVC. Remove it from the list of selectable words in the LVC;
2) – Select one word W2 outside of the LVC but with the same degree of W1. Remove it from the list of selectable words in the LVC. If no word with the same degree is selectable, discard W1 and repeat 1).

These two steps are iterated until X words have been sampled within and outside of the LVC with the same microscopic degree distribution (i.e. the same sequences of degrees in a given layer). For reducing fluctuations in the sampled averages due to finite size effects, we considered X = 1000 in this work. For reducing correlation effects between a word feature F and degree, we considered degrees of words in the layer having the highest Pearson's correlation between F and its degrees.

15. A Reduced Growth Model Induced by Concreteness Scores

In the main text, Figure 2a does not show a curve relative to concreteness scores, although concreteness is extensively discussed in the main text. However, Figure 2b complements Figure 2a in showing that concreteness scores are not the main linguistic feature influencing the emergence and the features of the LVC. The comparison indicates that polysemy scores display larger differences than concreteness when words within and outside of the LVC are compared. We could not include a model of lexical growth based on concreteness in Figure 2a because for over 600 words in the multiplex lexical representation there was no concreteness score available from the datasets. Rather than reducing the set of words in the multiplex representation, for which many additional features were indeed available, we performed the experiments reported in Figure 2b and then the statistical comparisons reported in Figure 3 and in the above Table.

As an additional check, we produced numerical experiments where we test a reduced version of our multiplex lexical network using the subset of words for which concreteness scores are available. In Figure S10, we compare results relative to letting this subgraph grow according to words inserted with higher polysemy/concreteness first against the normative case. While all the three cases display an explosive transition, the appearance of the LVC is delayed by roughly 2 years when more concrete words are inserted first in the multiplex lexical network. This further contributes to the message from the main text that concreteness is not the most prominent linguistic feature of words driving the LVC emergence.

In order to avoid confusion, Figure 2(b) refers to the larger multiplex structure with 8531 words, where the appearance of the LVC in time is fixed to the normative case. In Figure S10, instead time is not fixed but it is rather dependent on the growth dynamics determined by the concreteness scores.

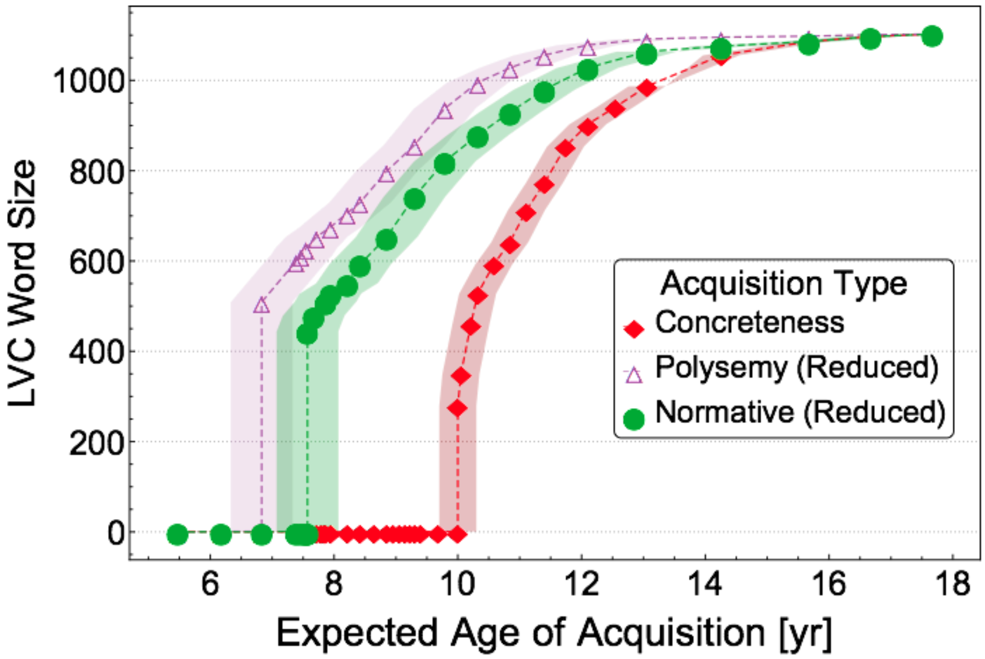

Supplementary Figure S10 : Lexical growth experiments similar to those reported in Figure 2 (a) of the main text but referring to a smaller multiplex structure including 618 words less compared to the one analysed in the main text. This reduction is induced by concreteness scores, which are not available for all 8531 words in the multiplex lexical representation but rather for only 7913 of them. Words with higher concreteness/polysemy are inserted first in the growing multiplex. The size of the LVC is then computed over the growing dynamics. Explosive transitions are evident in both the normative case, based on the empirical age of acquisition of words, and in concreteness/polysemy-based curves. Averages are relative to 50 independent iterations. Compared to the normative case, the appearance of the LVC is delayed by almost 2 years when concreteness scores are considered.

16. A Distance for Quantifying the LVC Growth Across Different Models

In order to compare the whole models of LVC evolution across development, we also consider the 1-norm distance s from the normative LVC size-trajectory, i.e. the sum of the absolute values of the LVC size in the normative case (LVC_{Norm}) minus the LVC size for a model (LVC_{Model}) based on frequency or concreteness, etc.. In formulas:

$$s_{Model} = \sum_t |LVC_{Norm.}(t) - LVC_{Model}(t)|$$

As the LVC size is measured in terms of words, the distance s is measured in words as well. Smaller distances indicate a larger agreement in the size of the LVC between a specific growth model and the normative case.

Supplementary Table S5 reports the distances for the different models tested in the main text. The closest model to normative acquisition in the frequency-based one, which however displays one more explosive transition on the evolution of the LVC size compared to the normative case. The second closest model to normative acquisition is the one based on polysemy score, while all other models based on word length, degree and random acquisition are more than 3 standard deviations away.

Model	Distance s from normative case (words)
Frequency	3750(30)
Random	8670(50)
Degree	11620(30)
Polysemy Scores	7110(50)
Word Length	8620(40)

Supplementary Table S5 : Distances for individual acquisition models of the LVC size evolution against the normative learning case. Error bars are reported in parenthesis and represent standard deviations based on 50 independent iterations of the LVC growth.

REFERENCES

1. Newman, M. Networks: an introduction. 2010. *United Slates: Oxford University Press Inc., New York*, 1-2.
2. Vitevitch, M. S. (2008). What can graph theory tell us about word learning and lexical retrieval?. *Journal of Speech, Language, and Hearing Research*, *51*(2), 408-422.
3. Steyvers, M., & Tenenbaum, J. B. (2005). The Large-scale structure of semantic networks: Statistical analyses and a model of semantic growth. *Cognitive science*, *29*(1), 41-78.
4. Vitevitch, M. S., Chan, K. Y., & Goldstein, R. (2014). Insights into failed lexical retrieval from network science. *Cognitive psychology*, *68*, 1-32.
5. Watts, D. J., & Strogatz, S. H. (1998). Collective dynamics of 'small-world'networks. *Nature*, *393*(6684), 440-442.
6. Beckage, N., Smith, L., & Hills, T. (2011). Small worlds and semantic network growth in typical and late talkers. *PloS one*, *6*(5), e19348.
7. Kiss, G. R., Armstrong, C. A., & Milroy, R. (1972). *An associative thesaurus of English*. Medical Research Council, Speech and Communication Unit, University of Edinburgh, Scotland.
8. Amancio, D. R., Oliveira Jr, O. N., & da Fontoura Costa, L. (2012). Identification of literary movements using complex networks to represent texts. *New Journal of Physics*, *14*(4), 043029.
9. Kenett, Y. N., Kenett, D. Y., Ben-Jacob, E., & Faust, M. (2011). Global and local features of semantic networks: Evidence from the Hebrew mental lexicon. *PloS one*, *6*(8), e23912.
10. Benedek, M., Kenett, Y. N., Umdasch, K., Anaki, D., Faust, M., & Neubauer, A. C. (2017). How semantic memory structure and intelligence contribute to creative thought: a network science approach. *Thinking & Reasoning*, *23*(2), 158-183.
11. Luce, P. A., & Pisoni, D. B. (1998). Recognizing spoken words: The neighborhood activation model. *Ear and hearing*, *19*(1), 1.
12. Chan, K. Y., & Vitevitch, M. S. (2010). Network structure influences speech production. *Cognitive Science*, *34*(4), 685-697.

13. Vitevitch, M. S., Chan, K. Y., & Roodenrys, S. (2012). Complex network structure influences processing in long-term and short-term memory. *Journal of memory and language*, *67*(1), 30-44.
14. Stella, M., Beckage, N. M., & Brede, M. (2017). Multiplex lexical networks reveal patterns in early word acquisition in children.*Scientific Reports*, *7*.
15. Sigman, M., & Cecchi, G. A. (2002). Global organization of the Wordnet lexicon. *Proceedings of the National Academy of Sciences*, *99*(3), 1742-1747.
16. Solonchak, T., & Pesina, S. (2015). Lexicon Core and Its Functioning. *Procedia-Social and Behavioral Sciences*, *192*, 481-485.
17. Xu, F., & Tenenbaum, J. B. (2007). Word learning as Bayesian inference. *Psychological review*, *114*(2), 245.
18. Ninio, A. (2014). Syntactic networks, do they contribute valid information on syntactic development in children?. Comment on" Approaching human language with complex networks" by J. Cong and H. Liu. *Physics of life reviews*, *11*, 632-634.
19. Battiston, F., Nicosia, V., & Latora, V. (2017). The new challenges of multiplex networks: measures and models. *The European Physical Journal Special Topics*, *226*(3), 401-416.
20. De Domenico, M., Nicosia, V., Arenas, A., & Latora, V. (2015). Structural reducibility of multilayer networks. *Nature communications*, *6*.
21. De Domenico, M., Solé-Ribalta, A., Cozzo, E., Kivelä, M., Moreno, Y., Porter, M. A., ... & Arenas, A. (2013). Mathematical formulation of multilayer networks. *Physical Review X*, *3*(4), 041022.
22. Warriner, A. B., Kuperman, V., & Brysbaert, M. (2013). Norms of valence, arousal, and dominance for 13,915 English lemmas.*Behavior research methods*, *45*(4), 1191-1207.
23. Aitchison, J. (2012). *Words in the mind: An introduction to the mental lexicon*. John Wiley & Sons.
24. Carlson, M. T., Sonderegger, M., & Bane, M. (2014). How children explore the phonological network in child-directed speech: A survival analysis of children’s first word productions.*Journal of memory and language*, *75*, 159-180.
25. Storkel, H. L. (2004). Do children acquire dense neighborhoods? An investigation of similarity neighborhoods in lexical acquisition. *Applied Psycholinguistics*, *25*(2), 201-221.
26. Storkel, H. L. (2009). Developmental differences in the effects of phonological, lexical and semantic variables on word learning by infants. *Journal of child language*, *36*(2), 291-321.
27. Garlock, V. M., Walley, A. C., & Metsala, J. L. (2001). Age-of-acquisition, word frequency, and neighborhood density effects on spoken word recognition by children and adults. *Journal of Memory and language*, *45*(3), 468-492.
28. Munson, B., Kurtz, B. A., & Windsor, J. (2005). The influence of vocabulary size, phonotactic probability, and wordlikeness on nonword repetitions of children with and without specific language impairment. *Journal of Speech, Language, and Hearing Research*, *48*(5), 1033-1047.
29. Newman, R. S., & German, D. J. (2002). Effects of lexical factors on lexical access among typical language-learning children and children with word-finding difficulties. *Language and Speech*,*45*(3), 285-317.
30. Rodd, J. M., Gaskell, M. G., & Marslen-Wilson, W. D. (2004). Modelling the effects of semantic ambiguity in word recognition.*Cognitive Science*, *28*(1), 89-104.
31. Keuleers, E., Lacey, P., Rastle, K., & Brysbaert, M. (2012). The British Lexicon Project: Lexical decision data for 28,730 monosyllabic and disyllabic English words. *Behavior Research Methods*, *44*(1), 287-304.
32. Feng, S., Cai, Z., Crossley, S. A., & McNamara, D. S. (2011, March). Simulating Human Ratings on Word Concreteness. In *FLAIRS Conference*.

33. Casas, B., Català, N., Ferrer-i-Cancho, R., Hernández-Fernández, A., & Baixeries, J. (2016). The polysemy of the words that children learn over time. *arXiv preprint arXiv:1611.08807*.
34. Lioma, C., Blanco, R., Palau, R. M., & Moens, M. F. (2009). A Belief Model of Query Difficulty That Uses Subjective Logic. *ICTIR*, *5766*, 92-103.
35. Kuperman, V., Stadthagen-Gonzalez, H., & Brysbaert, M. (2012). Age-of-acquisition ratings for 30,000 English words. *Behavior Research Methods*, *44*(4), 978-990.
36. Solé, R. V., & Seoane, L. F. (2015). Ambiguity in language networks. *The Linguistic Review*, *32*(1), 5-35.
37. Stella, M., & Brede, M. (2015). Patterns in the English language: phonological networks, percolation and assembly models. *Journal of Statistical Mechanics: Theory and Experiment*, *2015*(5), P05006.
